# Mapping OMIM Disease–Related Variations on Protein Domains Reveals an Association Among Variation Type, Pfam Models, and Disease Classes

**DOI:** 10.3389/fmolb.2021.617016

**Published:** 2021-05-07

**Authors:** Castrense Savojardo, Giulia Babbi, Pier Luigi Martelli, Rita Casadio

**Affiliations:** ^1^Biocomputing Group, Department of Pharmacy and Biotechnology, University of Bologna, Bologna, Italy; ^2^Institute of Biomembranes, Bioenergetics and Molecular Biotechnologies, National Research Council, Bari, Italy

**Keywords:** protein variations, protein structure, protein domain, variation type, disease-related variations, disease variant databases, Pfam-disease association

## Abstract

Human genome resequencing projects provide an unprecedented amount of data about single-nucleotide variations occurring in protein-coding regions and often leading to observable changes in the covalent structure of gene products. For many of these variations, links to Online Mendelian Inheritance in Man (OMIM) genetic diseases are available and are reported in many databases that are collecting human variation data such as Humsavar. However, the current knowledge on the molecular mechanisms that are leading to diseases is, in many cases, still limited. For understanding the complex mechanisms behind disease insurgence, the identification of putative models, when considering the protein structure and chemico-physical features of the variations, can be useful in many contexts, including early diagnosis and prognosis. In this study, we investigate the occurrence and distribution of human disease–related variations in the context of Pfam domains. The aim of this study is the identification and characterization of Pfam domains that are statistically more likely to be associated with disease-related variations. The study takes into consideration 2,513 human protein sequences with 22,763 disease-related variations. We describe patterns of disease-related variation types in biunivocal relation with Pfam domains, which are likely to be possible markers for linking Pfam domains to OMIM diseases. Furthermore, we take advantage of the specific association between disease-related variation types and Pfam domains for clustering diseases according to the Human Disease Ontology, and we establish a relation among variation types, Pfam domains, and disease classes. We find that Pfam models are specific markers of patterns of variation types and that they can serve to bridge genes, diseases, and disease classes. Data are available as Supplementary Material for 1,670 Pfam models, including 22,763 disease-related variations associated to 3,257 OMIM diseases.

## Introduction

In the last decade, several efforts have been devoted to the problem of functional annotation of protein variants with the aim of relating variations to specific diseases (Vihinen, 2017, 2018). A collection of variations of genetic diseases is now available, and this prompted the investigation of molecular mechanisms responsible for protein failure (Schaafsma and Vihinen, 2018). Particularly, variations of non-synonymous proteins can promote the change of the active/binding sites and/or protein instability and can hamper protein–protein and ligand–protein interactions (Kucukkal et al., 2015; Ittisoponpisan et al., 2019; Ofoegbu et al., 2019). Molecular mechanisms can be, therefore, different, and different phenotypes may share common molecular mechanisms, independent of the different genes (Deans et al., 2015; Reeb et al., 2016; Babbi et al., 2019, and references therein). Several studies also focused on determining the most frequent protein variants associated with diseases, with the aim of helping functional annotation, starting from variant sequencing (Niroula and Vihinen, 2017; Zeng and Bromberg, 2019).

Different computational methods are available for the functional annotation of variations, based on different approaches. Routinely, given a specific variation, computational methods return with a computed reliability whether the change of a side chain in a protein is disease-related or not (Niroula and Vihinen, 2016).

An interesting aspect of disease-related protein variants is the protein instability promoted by the variations (Casadio et al., 2011; Savojardo et al., 2019, and references therein). Protein instability may be related to a disease, with this not being the only reason. For functional annotation of disease-related variations, routinely, the chemico-physical properties of the variation and the effect of the variation on the close environment in the protein structure are taken into consideration. It appears that the correlation among the strength of association to disease and the strength of association to the protein structure perturbation is moderate (Savojardo et al., 2019).

The problem of which phenotype is associated with a given variation or a set of variations has been scarcely addressed, and it remains unanswered, given the complexity of the scenario relating phenotypes to variations. Existing databases can relate genes to diseases and/or variations to diseases (MalaCards^1^, Rappaport et al., 2017; GeneCards^2^, Stelzer et al., 2016; DisGeNet^3^, Piñero et al., 2020; eDGAR^4^, Babbi et al., 2017; Humsavar^5^, UniProt Consortium, 2019; OMIM^6^, Amberger et al., 2015).

Protein domains have been adopted to explore associations between genes and human-inherited diseases (Zhang et al., 2011, 2016; Yates and Sternberg, 2013; Wiel et al., 2017, 2019). Models of protein domains are available in the Pfam database^7^ (El-Gebali et al., 2019), and they enable the clustering of proteins into protein families, each represented by multiple sequence alignments, mainly based on protein structural alignments and cast into hidden Markov models (HMMs). Initially, similarities of disease phenotypes were exploited within a given domain–domain interaction network, and a Bayesian approach was proposed to prioritize candidate domains for human complex diseases (Zhang et al., 2011). Then, domain–disease associations were inferred from domain–protein, protein–disease, and disease–disease relationships (Zhang et al., 2016). In these studies, the bottom layer of variations in proteins, detected in large-scale sequencing experiments, was not taken into consideration, restraining the analysis only to the already known protein– or gene–disease associations. More recently (Wiel et al., 2017), with the notion of homologous domains in proteins, variants were aggregated to improve their interpretation, and a web server (MetaDome^8^, Wiel et al., 2019) was made available for the pathogenicity analysis of genetic variants.

In a previous study (Savojardo et al., 2019), we introduced the notion of variation type, in order to take the physico-chemical properties of the variations into account as well (Casadio et al., 2011). After mapping genetic disease–related variations on a restricted set of human protein three-dimensional (3D) structures, we found that the distribution of disease variation types significantly varies across different structural/functional Pfam models.

In this study, relying on the relationship between genes and phenotypes, we ask the question as to which extent possible patterns of variation types framed into Pfam domains are significant for a reliable association to specific groups of maladies.

## Materials and Methods

### Dataset Construction

The dataset adopted in this study was derived from the Humsavar database^5^ release 2020_04 of August 2, 2020, listing all missense variants annotated in human UniProtKB/Swiss-Prot (UniProt Consortium, 2019) entries.

From the initial set of proteins included in the database, we only selected those reporting at least one variant implicated in the disease, excluding proteins reporting only polymorphisms not associated with disease insurgence. Moreover, any variation labeled as ‘‘unclassified’’ (i.e., with uncertain implications in disease) was filtered out. Finally, we only retained disease-related variations associated with a genetic disorder reported in the Online Mendelian Inheritance in Man (OMIM) catalog^9^.

The set of neutral variations was extended using data retrieved from the GnomAD database (exome version 2.1.1) (Karczewski et al., 2020). Only variations occurring in our set of proteins, not already included in Humsavar and with clinical significance labeled as “Benign/Likely benign” by ClinVar (release 2021-03-23) (Landrum et al., 2020), were retained.

Pfam (El-Gebali et al., 2019) annotations were retrieved from the Pfam-A region annotation file for *Homo sapiens* version 33.1 obtained *via* the Pfam FTP server^10^. From all the annotations available, we only retained those occurring at proteins included in our set of data and covering at least one disease-related variation.

### Mapping OMIMs to Disease Ontology

The DO (Human Disease Ontology) OBO (Open Biological and Biomedical Ontology) file release of September 15, 2020, was downloaded^11^ and used directly to retrieve annotations for each OMIM disease by means of cross-references. Each retrieved leaf DO term associated to a single OMIM was expanded up to the ontology root term, including all ancestors. Term expansion was computed using an *ad-hoc* script to parse the OBO file.

### Computing the Disease Score

For each Pfam domain, we estimated a propensity score for the association to the disease as follows:

(1) $$\mathrm{Score}\left(\mathrm{pfam}\right)=\frac{N_{d}^{\mathrm{pfam}}/\left(N_{d}^{\mathrm{pfam}}+N_{p}^{\mathrm{pfam}}\right)}{N_{d}/\left(N_{d}+N_{p}\right)}$$

where $N_{d}^{\mathrm{pfam}}$ and $N_{p}^{\mathrm{pfam}}$ are the number of disease-related and polymorphism variations in the domain *pfam*, while *N*_*d*_ and *N*_*p*_ are the same numbers in the whole dataset. In the dataset, scores range from 1.40 down to 0.03.

### Kullback–Leibler Divergence Between Distributions

Differences between probability distributions were evaluated using the Kullback–Leibler divergence:

(2) $$D_{KL}=-\sum_{x\in X}p\left(x\right)\cdot log_{2}\frac{q\left(x\right)}{p\left(x\right)}$$

where *p* and *q* are two discrete probability distributions defined on the same probability space *X*.

## Results

### A Dataset of Variations With Annotated Pfam

Overall, our dataset comprises 50,746 variations occurring in 2,959 proteins implicated in 3,884 genetic disorders. Disease-related variations in these proteins are 29,949, accounting for 55% of the total variations. The remaining 20,797 variations are neutral (45%). Table 1 shows summary statistics about the dataset analyzed in this study.

**TABLE 1:** Summary of the OMIM-related variation dataset of this study.

Number of proteins associated with disease	2959
Number of diseases (OMIM)	3884
Number of variations	54746
Number of disease variations	29949 (55%)^
Number of neutral polymorphisms (on the same disease proteins)	24797 (45%)^
Number of disease proteins with Pfam covering disease variations	2513 (85%)^#^
Number of Pfams	1670
Number of diseases (OMIM) in proteins with Pfams	3257 (84%)°
Number of variations covered by Pfams	31934 (68%)*^*
Number of disease variations covered by Pfams	22763 (71%)^+^
Number of neutral polymorphisms covered by Pfams	9171 (29%)^+^

*^ percentage computed with respect to the total number of variations (54746); ^#^ percentage computed with respect to the total number of proteins (2959); ° percentage computed with respect to the total number of diseases (3884); ^+^ percentage computed with respect to the total number of Pfam-covered variations (31934).*

Restricting the set of proteins to those having Pfam entries covering at least one disease-related variation, we ended up with 2,513 proteins (corresponding to 85% of the initial protein set) implicated in 3,257 distinct genetic diseases. Overall, 1,670 distinct Pfam entries were annotated on these proteins. A subset of 548 out of 1,670 Pfams occurs in two or more proteins in the set. The vast majority (96%) of Pfam entries are of type “Domain” or “Family,” while a very small fraction accounts for “Repeat,” “Coiled-coil,” “Motif,” and “Disordered” types.

After this reduction, we retained 31,934 variations covered by Pfams, distributed into 22,763 (71%) and 9,171 (29%) disease-related and neutral polymorphic variations, respectively.

Data shown in Table 1 clearly indicate that the incidence of disease-related variations within Pfam domains is significantly higher than the background (71% against 55%).

### Overall Pfam Association With Disease

We were interested in elucidating the overall association between Pfam and OMIM diseases. For each entry in the set of 1,670 Pfam domains in our dataset, we computed the score for the association to disease with the formula reported in Eq. 1. A value greater than 1 for this ratio highlights a higher abundance of disease variations in the Pfams than in the background. The complete result of this analysis is reported in Supplementary Table 1 for all the 1,670 Pfam entries. About 48% of Pfam entries have a value greater than 1, as a consequence of the overall propensity of disease-related variations to be located within Pfam domains. In general, the distribution of scores is not random and reflects a differential disease association for the different Pfam entries.

In Table 2, we list the result for the 20 highest scoring Pfams covering 10 or more proteins. Scores with corrected *p*-values (Supplementary Table 2) equal to or lower than 0.1 are highlighted (top scoring Pfams are all significant at 0.1 level). Significance does not hold for some Pfams covering only few variations. In these cases, more data are needed in order to properly evaluate the association to the disease.

**TABLE 2:** The 20 highest scoring Pfam entries mostly associated with diseases.

Pfam ID	Pfam name	Pfam type	No of proteins	No of disease variations	No of neutral polymorphisms	Score^§^
PF00105	zf-C4	Domain	12	60	2	1.36*
PF00250	Forkhead	Domain	10	88	4	1.34*
PF00010	HLH	Domain	14	48	3	1.32*
PF00104	Hormone_recep	Domain	18	195	20	1.27*
PF00307	CH	Domain	11	48	6	1.25*
PF00046	Homeodomain	Domain	42	163	21	1.24*
PF07645	EGF_CA	Domain	17	301	46	1.22*
PF00096	zf-C2H2	Domain	23	80	13	1.21*
PF00029	Connexin	Family	10	319	53	1.20*
PF00017	SH2	Domain	11	72	12	1.20*
PF00520	Ion_trans	Family	48	1020	173	1.20*
PF00004	AAA	Domain	10	70	12	1.20*
PF00400	WD40	Repeat	19	52	9	1.20
PF02770	Acyl-CoA_dh_M	Domain	10	40	7	1.19
PF00169	PH	Domain	11	53	10	1.18
PF00005	ABC_tran	Domain	15	236	49	1.16*
PF07686	V-set	Domain	12	84	18	1.16
PF00271	Helicase_C	Family	17	65	15	1.14
PF00176	SNF2_N	Family	10	63	15	1.13
PF00089	Trypsin	Domain	21	258	87	1.13*

^§^Score is computed as defined in Eq. 1. Significance of each score was assessed using the Fisher exact test on the corresponding contingency table and correcting for multiple testing using the Benjamini-Hochberg procedure. Individual p-values are listed in Supplementary Table 2. *Corrected P-values are equal or lower than 0.1.

Interestingly, Pfam entries reported in Table 2 can be grouped into few functional classes, including DNA-binding domains (accounting for eight domains/families), transmembrane domains (three), and enzymes (three).

### Pfams Have Distinctive Patterns of Disease Variation Types

**FIGURE 1:**
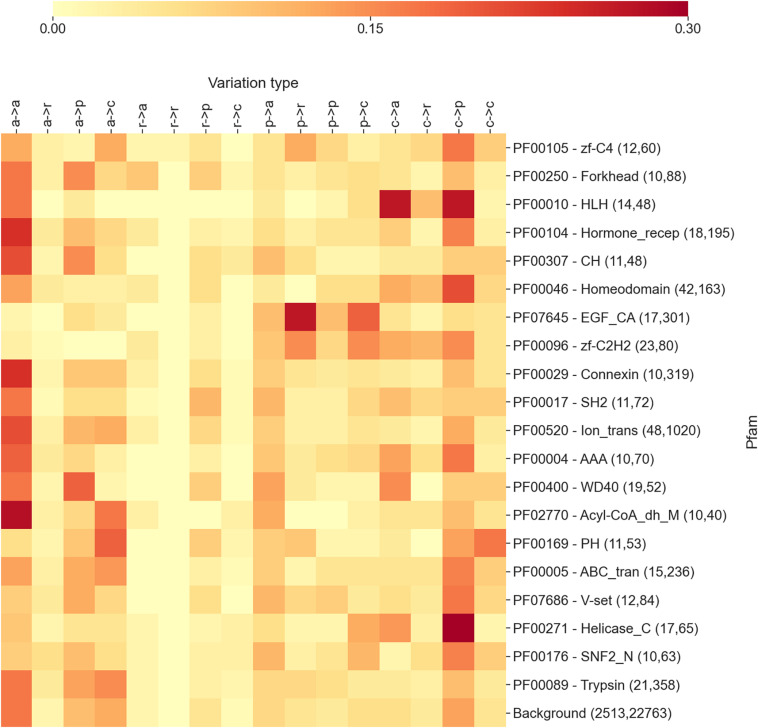
The heatmap reporting the frequency of each variation type as observed within the 20 Pfam entries mostly associated with diseases. For each Pfam, the numbers within parentheses indicate the number of proteins and disease-related variations covered. In variation types, labels are as follows: a, apolar; r, aromatic; p, polar; and c, charged. Mean and median Kullback–Leibler divergences (Eq. 2) between individual Pfam distributions and the background are 2.1 and 2.1 bits, respectively.

Going a step further in the analysis, we investigated the composition of disease-related variations occurring in different Pfam domains. In a previous study (Savojardo et al., 2019), the same analysis was performed on a small dataset of highly curated variations covered by 3D structures from Protein Data Bank (PDB). In this study, we extended and complemented the previous results using a larger dataset of Pfam domains and variations. To this aim, we first grouped residues according to their physico-chemical properties, obtaining four major groups, namely, apolar (GAVPLIM), aromatic (FWY), polar (STCNQH), and charged (DEKR) residues. We define a variation type in relation to the conservation or substitution of apolar (a), polar (p), aromatic (r), and charged (c) (Figure 1). Then, we computed Pfam-specific distributions of disease-related variations involving substitutions from one group to another (overall, 16 different substitution types are possible). Complete results are reported in Supplementary Table 3 for all the 1,670 Pfam domains.

In Figure 1, we show a heatmap reporting the frequencies of each substitution type for the 20 highest scoring Pfam entries described in the previous section and mostly associated with diseases. For each Pfam entry, we report the Pfam ID, the name, and two numbers in parentheses, indicating the number of proteins and disease-related variations covered by the specific Pfam. For comparison, the last row reports the overall distribution of substitution types computed on the whole set of variation types covered by Pfams.

The results shown in the heatmap of Figure 1 indicate that the different Pfams are enriched in different variation types and that each Pfam shows a differential pattern with respect to the background. Interestingly, in some cases, the pattern of enriched variation types can be related with the overall function of the Pfam domain and/or the cellular context in which the domain/s are presumably operating.

**FIGURE 2:**
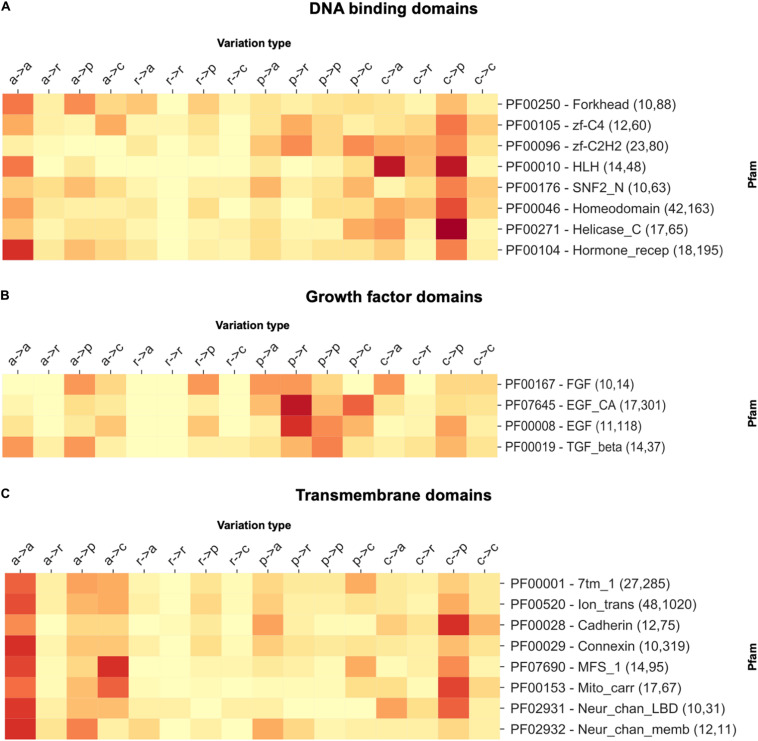
The heatmap reporting the frequency of each variation type as observed within a selection of **(A)** DNA-binding, **(B)** growth factor, and **(C)** transmembrane domains. For each Pfam, the numbers within parentheses indicate the number of proteins and disease-related variations covered. In variation types, labels are as follows: a, apolar; r, aromatic; p, polar; and c, charged.

In Figure 2, we report three examples, namely, a selection of DNA-binding domains, growth factors, and transmembrane domains. For DNA-binding domains, we observe a higher concentration of disease-related variations involving a substitution from a charged residue to any different residue type. Contrarily, for growth factor domains, we observe abundant variations involving substitutions from polar to any type of the residue, while transmembrane domains are mostly enriched in substitutions involving apolar wild types. These observations clarify a general trend, pointing to the specificity of the disease variation type per Pfams of functional classes.

From data analysis, we conclude that the distribution of the disease-related variation type patterns observed for the different Pfams is non-random and different from the background distribution (computed considering all the disease-related variation types occurring in Pfams). This observation confirms our previous results obtained with a smaller number of Pfam domains, directly related to human protein structures, and corroborates the notion that distinctive patterns of disease-related variation types are Pfam specific (Savojardo et al., 2019).

### Linking the Pfam to Disease Ontology

As a final step of our investigation, we searched for a link between Pfam domains and disease ontology. Disease classification is not a trivial task. Different controlled vocabularies and ontologies such as the Human Phenotype Ontology (HPO)^12^ (Köhler et al., 2019) or the DO (Schriml et al., 2019) are available for this purpose. However, none of the ontologies provides a full coverage of the entire space of OMIM diseases, ranging from 82% coverage of HPO to 74% of DO. Moreover, ontologies like HPO are not specifically designed to describe a disease. Instead, they are devised to describe clinically relevant phenotypes. In the current study, we used the DO ontology because, in spite of a slightly lower coverage, it provides a better and less ambiguous classification of diseases.

To obtain a high-level disease classification, we collected all the 3,257 OMIM diseases linked to variations occurring in our 1,670 Pfam domains and mapped them to a set of 17 first-level DO terms. These include 12 terms describing diseases affecting anatomical entities (all child terms of “DOID:7 – disease of anatomical entity” like cardiovascular, endocrine, gastrointestinal, etc.), cellular proliferation diseases (DOID:14566), mental health diseases (DOID:150), metabolic diseases (DOID:0014667), physical disorders (DOID:0080015), and syndromes (DOID:225). We were able to map 2,454 out of 3,257 OMIMs to at least one of the above DO terms. On average, each OMIM was mapped to 1.01 DO, providing an almost strict classification of each OMIM into a single DO term.

With this mapping, we computed a Pfam-specific distribution of DO-associated disease classes. Complete results are reported in Supplementary Table 4 for all the 1,670 Pfam entries considered in this study. The data provided in this study indicate that disease classes are not evenly distributed among different Pfam domains, again suggesting a differentiated association between the Pfam and phenotypes.

**FIGURE 3:**
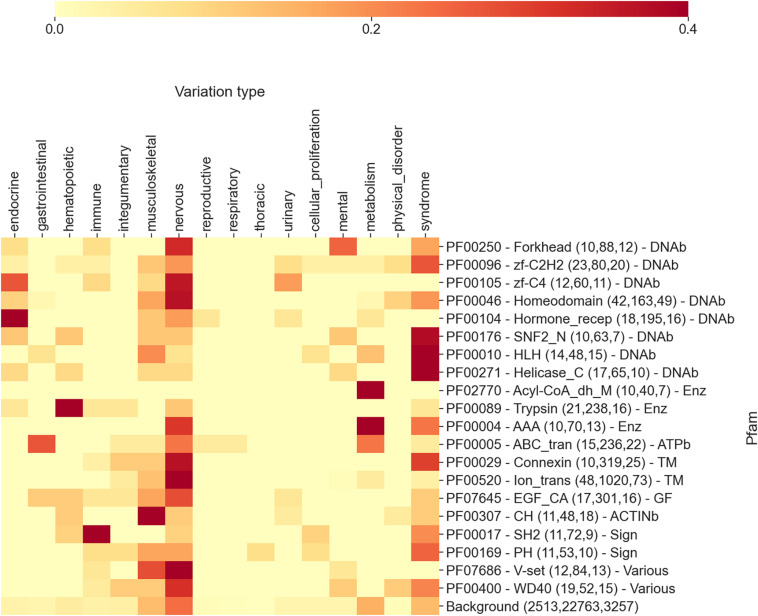
The heatmap reporting, for each Pfam, the frequency of diseases (grouped into 17 different classes extracted from Disease Ontology) as retrieved from OMIMs, after the association *via* the disease type with Pfam. The numbers within parentheses are the number of proteins, the number of disease variations, and the number of Online Mendelian Inheritance in Man (OMIM) diseases associated with the Pfam, respectively. Each Pfam is labeled according to its functional class: DNAb, DNA-binding domain; Enz, enzymatic domain; TM, transmembrane; GF, growth factor; ACTINb, actin-binding domain; Sign, signaling; and Various, various functions associated. Mean and median Kullback–Leibler divergences (Eq. 2) between individual Pfam distributions and the background are 2.5 and 2.7 bits, respectively.

In Figure 3, we show an extract of our analysis, focusing on the 20 highest scoring Pfam domains associated with diseases. The heatmap reports, for each Pfam, the frequency of disease types (in the 17 different classes detailed above) as retrieved from OMIMs associated with substitutions occurring on the specific Pfam. In brackets, close to each Pfam name, we list the number of proteins, disease variations, and OMIMs associated to the Pfam.

Even in this case, the distributions of disease classes appear to be very different from the background (reported in the last row of the heatmap). Remarkably, the aggregation of Pfams into more general functional classes provides an additional level of interpretation. Considering Figure 3, we can observe that DNA-binding domains are mostly associated with syndromes, nervous system, and endocrine system disease classes, while enzymes are mostly involved in the metabolic disease class. Transmembrane domains show the prevalence of nervous and integumentary disease classes, while growth factors and actin-binding domains are enriched in musculoskeletal diseases. Finally, signaling Pfam domains are prominently associated with immune system diseases. Overall, many of these findings are in line with what we expected. Protein domains have different functions and are involved into different biological processes. Variations occurring in these domains, when disruptive, lead to diseases that are connected to the biological processes in which the proteins are mainly involved. For instance, the fact that variations occurring in transmembrane domains are often linked to neurological diseases is a direct consequence of the involvement of transmembrane proteins (among other functions) in neurotransmission. Similarly, variations in enzymes routinely lead to metabolic diseases.

**FIGURE 4:**
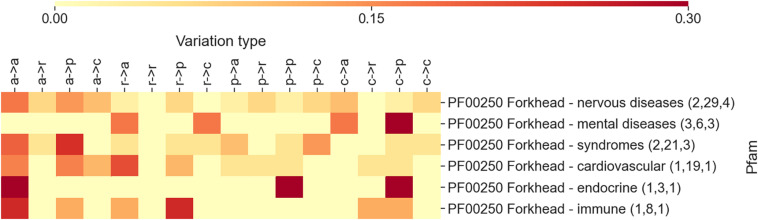
The heatmap reporting, for the Pfam entry PF00250 Forkhead, the frequency of each variation type as observed after separating variations according to disease classes. The numbers within parentheses are the number of proteins, the number of disease variations, and the number of Online Mendelian Inheritance in Man (OMIM) diseases associated with the Pfam (and covered by DOID), respectively.

Some of the Pfams reported in Figure 3 are associated to more than one disease types. For example, diseases that are associated to the Forkhead domain (PF00250) are distributed into five classes, namely, nervous, mental, endocrine, immune diseases, and syndromes. In Figure 4, an additional heatmap is shown trying to link the disease types to the patterns of variation types. Specifically, the patterns of variation types are reported after isolating variations linked to OMIMs in the different disease classes. Interestingly, the patterns show an evident difference among each other. This confirms the level of association that links domains to variation types and diseases.

## Conclusion and Perspectives

In this study, we consider, for the time being, only diseases of genetic origins, with the belief that cancer-related somatic variations are as yet not satisfactorily clustered according to tissue specificity of the plague.

This study, as well as the previous ones (Yates and Sternberg, 2013; Wiel et al., 2017, 2019), aims at establishing a direct mapping among variations, diseases, and phenotypes *via* the protein domains. Our novelty is the introduction of the variation type as a distinguished feature of association to the Pfam domain and to the phenotype. Our findings complement previous ones (Wiel et al., 2017) with the inclusion of the variation type, which adds to the classification of variations and their impact on the protein function, stability, and interaction in the specific context where the gene is active.

The link among the variation type, Pfam domain, and phenotype can greatly reduce the number of possible steps to understand which variations are disease-related or which are not and which phenotype they may promote. In perspective, the association among the variation type, protein domain/s, and phenotype may greatly simplify the problem of genetic variant annotation.

## Data Availability Statement

The original contributions presented in the study are included in the article/Supplementary Material, further inquiries can be directed to the corresponding author.

## Author Contributions

RC, PM, and CS: conceptualization, methodology, and writing. CS: software. GB and CS: data curation and visualization. RC and PM: supervision. All authors contributed to the article and approved the submitted version.

## Conflict of Interest

The authors declare that the research was conducted in the absence of any commercial or financial relationships that could be construed as a potential conflict of interest.

## Footnotes

1 https://www.malacards.org/

2 https://www.genecards.org/

3 https://www.disgenet.org/

4 http://edgar.biocomp.unibo.it

5 https://www.uniprot.org/docs/humsavar

6 https://www.omim.org/

7 https://pfam.xfam.org/

8 https://stuart.radboudumc.nl/metadome/

9 https://omim.org/

10 ftp.ebi.ac.uk/pub/databases/Pfam/releases/Pfam33.1/proteomes/9606.tsv.gz

11 https://disease-ontology.org/

12 https://hpo.jax.org/app/
