# Supplementary material for: Mapping OMIM Disease–Related Variations on Protein Domains Reveals an Association Among Variation Type, Pfam Models, and Disease Classes
Source: Front Mol Biosci. 2021 May 7;8:617016. doi: 10.3389/fmolb.2021.617016 (PMC8138129; doi:10.3389/fmolb.2021.617016)
Supplement: Supplementary file 2 [file Table_2.docx]

**Table S2**. Corrected P-values for the 20 top-scoring Pfams, assessed using a Fisher exact test on the corresponding contingency table and correcting for multiple testing using the Benjamini-Hochberg procedure.

| **Pfam ID** | **Pfam Name** | **Pfam Type** | **Corrected p-value** |
| --- | --- | --- | --- |
| PF00105 | zf-C4 | Domain | < 0.0001 |
| PF00250 | Forkhead | Domain | < 0.0001 |
| PF00010 | HLH | Domain | 0.002 |
| PF00104 | Hormone_recep | Domain | < 0.0001 |
| PF00307 | CH | Domain | 0.0334 |
| PF00046 | Homeodomain | Domain | < 0.0001 |
| PF07645 | EGF_CA | Domain | < 0.0001 |
| PF00096 | zf-C2H2 | Domain | 0.014 |
| PF00029 | Connexin | Family | < 0.0001 |
| PF00017 | SH2 | Domain | 0.029 |
| PF00520 | Ion_trans | Family | < 0.0001 |
| PF00004 | AAA | Domain | 0.04 |
| PF00400 | WD40 | Repeat | 0.126 |
| PF02770 | Acyl-CoA_dh_M | Domain | 0.26 |
| PF00169 | PH | Domain | 0.183 |
| PF00005 | ABC_tran | Domain | 0.0002 |
| PF07686 | V-set | Domain | 0.106 |
| PF00271 | Helicase_C | Family | 0.32 |
| PF00176 | SNF2_N | Family | 0.393 |
| PF00089 | Trypsin | Domain | 0.0002 |
